# Updated nutritional program improves performance, carcass yield, and profitability in male and female broilers

**DOI:** 10.1016/j.psj.2025.106283

**Published:** 2025-12-13

**Authors:** Filipe A. Monteiro, Carlos H. Oliveira, Maria R.S. Farias, Lucimauro Fonseca, Bruno T. Ramos, Gabriel R. Braga, Sarah M. Andrade, Arele A. Calderano, Horacio S. Rostagno, Melissa I. Hannas

**Affiliations:** Department of Animal Science, Federal University of Viçosa, Viçosa, MG 36570-900, Brazil

**Keywords:** Broiler nutrition, Feeding strategies, Nutrient utilization, Nutritional requirements

## Abstract

A study was conducted to assess the impact of nutritional advancements on broiler production and economic profitability. A total of 1,600 broiler chickens were distributed into eight treatments, with ten replicates and 20 birds per experimental unit. The treatments followed a 2 × 4 factorial arrangement, with sex (male and female) and four nutritional programs (Brazilian Tables for Poultry and Swine - BT 2024, BT 2017, BT 1983, and Cobb 2022). Growth performance was assessed from 01 to 42 days of age. Carcass trait was assessed at 42 days of age. Body composition was measured using dual-energy X-ray absorptiometry (DXA) at 27 and 42 days of age. Data were submitted to ANOVA at a 5 % significance level, and differences were determined using the SNK test (P < 0.05). An economic analysis considered feed cost per bird, feed cost per kilogram of ADG, gross revenue, and net revenue per bird. Broiler performance, carcass traits, and body composition were influenced by the interaction between sex and nutritional program. Assessing the programs within each sex, for males and females, BT 2024 and BT 2017 improved performance, body composition, and carcass traits compared with BT 1983, with Cobb 2022 showing intermediate results. However, the lowest feed intake was observed in BT 2024, which resulted in the best feed conversion ratio. Fat content was lower in BT 2024. Assessing the sex within each program, males exhibited superior performance, carcass weight, and body composition, whereas females showed higher breast yield. Economic analysis indicated that BT 2024 achieved the highest net revenue due to lower feeding cost per kilogram of ADG, despite BT 2017 showing the greatest gross revenue. In conclusion, BT 2024 demonstrates superior performance and economic efficiency, indicating the importance of continuously updating nutritional recommendations.

## Introduction

Over the past decades, broiler production has transformed, evolving from small-scale activity into highly industrialized system driven by intensive genetic selection and nutrition research (Elwinger et al., 2016). This period has marked unprecedented genetic progress, with modern broiler strains exhibiting substantially higher growth rates, feed conversion efficiency, and breast meat yield than their predecessors (Zuidhof et al. 2014). However, the proportion of modern broiler performance gains that is attributable to advancements in nutrition formulation in the last decades was unknown, since Havenstein et al. (1994a, 1994b) identified that genetic improvement accounted for broiler weight gains by a factor of 3.5, while nutrition was responsible for 20% of the increase from a specific historical context (1957 until 1991).

The evolution of broiler nutrition has been defined by key principles that fundamentally transformed feeding strategies. Early nutritional research, intensifying from the 1940s and reaching a peak in the 1970s, focused primarily on identifying essential nutrients and establishing basic requirements (Elwinger et al., 2016). In this context, the publication of the first edition of Nutrient Requirements of Poultry by National Research Council (U.S) in 1954 established a foundational reference that guided global poultry nutrition. Subsequently, other feeding standards emerged, including Brazilian Tables for Poultry and Swine (BT), first published in 1983 based on locally conducted research. Since its inception, the Brazilian Tables has undergone five revisions (2000, 2005, 2011, 2017, and 2024), each incorporating contemporary research findings and adapting to the genetic potential of modern strains.

The nutritional advances implemented across these last 40 decades have been substantial and multifaceted and have been marked by changes in protein and amino acid (**AA**) management, energy adjustment, leading to the adoption of more precise feeding programs, etc. Nutritional requirements of broilers have evolved with the genetic progress of modern strains, which exhibit greater protein deposition capacity and higher demand for digestible AA, particularly lysine (**Lys**) (Sharma et al., 2018; Maharjan et al., 2020). Metabolizable energy (**ME**) has become the main reference, but recent studies have emphasized the digestible Lys to ME ratio (**dLys:ME**) as a critical parameter for performance and carcass yield (Barekatain et al., 2021; Mansilla et al., 2022; Toghyani et al., 2024). Other advances have been feeding broilers consider physiological changes along growth and sex separated. Phase program from simple two-phases to complex multi-phase systems (up to five phases), enabling closer alignment between nutrient supply and the dynamic physiological requirements throughout the growth cycle (Hauschild et al., 2015; Tikate et al., 2021). Sexual dimorphism in broiler chickens represents a critical consideration in modern poultry nutrition, with males and females exhibiting measurably different growth trajectories, feed conversion ratios, and nutrient requirements (England et al. 2022). Despite these documented performance and economic signals, commercial adoption of sex-specific phase feeding programs remains inconsistent, largely due to uncertainties regarding the cost-benefit balance across varying market conditions, production systems, and genetic lines (Da Costa et al. 2017; Pesti and Miller 2013).

As genetics has continually enhanced the growth potential, feed efficiency, and robustness of modern broilers, breeding companies provide updated nutritional recommendations to ensure that nutritional supply is aligned with the productive potential of latest strains (Cobb-Vantress, 2022). Additionally, poultry science operates on continuously actualization of feeding standards as exemplified by the five updates of to the Brazilian Tables science 1983. However, for over 40 years, the productive and economic return on the investment from these progressively complex nutritional programs and sex-separated has not been quantified in a controlled framework. While genetic progress enhances broiler potential, and research examines isolated nutrients, no study adopted a holistic, longitudinal perspective.

In this context, we hypothesized that the cumulative nutritional advancements implemented over four decades have been essential to access the genetic potential of modern broilers, and feeding contemporary strains according to updated versus historical nutritional programs would result in measurable improvements in growth performance, body composition, carcass characteristics, and economic profitability. Therefore, the objective of this study was to evaluate the growth performance, body composition (assessed via DXA), carcass traits, and economic profitability of male and female broilers from contemporary commercial strain (Cobb 500) fed according to four nutrition programs representing key milestones in the evolution of broiler nutrition over the past 41 years (Brazilian Tables 1983, 2017, 2024, plus Cobb 2022 recommendations), thereby quantifying the cumulative impact of nutritional advancements on productivity and economic returns in modern broiler production systems.

## Materials and methods

### Care and use of animals

All procedures performed in the study were in accordance with the guidelines of the Brazilian National Council for Animal Experimentation Control (CONCEA). The research protocol was approved by the Ethics Commission on the Use of Farm Animals of Federal University of Viçosa (CEUAP-UFV), under protocol no. 58/2024.

### Animal husbandry and experimental design

The experiment was conducted in the Unit for Teaching, Research, and Extension in Poultry and Laying Hens of the Department of Animal Science at Federal University of Viçosa. One-day-old broiler chickens (Cobb 500) were obtained from a commercial hatchery, having been vaccinated against Marek’s disease, Newcastle disease, and infectious bronchitis, in accordance with current sanitary protocols.

A total of 800 one-day-old male and 800 one-day-old female broiler chickens (n = 1,600), with an initial weight of 43.0 ± 0.125 g, were randomly allocated to eight treatments in a 2 × 4 factorial arrangement, with sex (male and female) and four nutritional programs (BT 2024, BT 2017, BT 1983, and Cobb 2022) being the treatments. Each treatment was replicated ten times, with each replicate consisting of concrete pens 0.6 m x 1.5 m x 2.0 m (height x width x length) housing 20 chicks (stocking density of 10 birds/m²) with one feeder, two drinking nipples cups and covered with fresh wood shavings as bedding material. Birds had free access to feed and water during the entire experimental period. The house environment was partially controlled using canopy-type electric brooders for heating with incandescent lamps (250 W) used as heaters in each pen from 1 to 14 days and curtain management for airflow, and fans for cross ventilation (positive pressure) after 14 until 42 days. The temperature was monitored daily at 7 h, 10 h, 12 h, 15 h, and 18 h, using digital thermometers placed at bird height. The environmental temperature ranged at minimum and maximum from 22.6 to 31.9, 22.3 to 31.0 and 22.6 to 30.2 °C from 1 to 14, 14 to 28 and 28 to 42 d, respectively. The lighting program consisted of 24 hours of light for the first 14 days whereafter the photoperiod was decreased to 18 hours of light and 6 hours of dark.

### Experimental diets

The treatments consisted of four nutritional programs for each sex (8 treatments; Table 1). The BT 2024 and 2017 diets were formulated separately for males and females to fully meet the requirements of high-performance broilers using five and four phase feeding programs, according to Hannas et al. (2024) and Rostagno et al. (2017), respectively. In contrast, the BT 1983 diets were formulated as a single diet to meet the requirements of both sexes in a two-phase feeding program, according to Rostagno et al. (1983). Similarly, the Cobb 2022 diets were formulated as a single diet to meet the requirements of both sexes, in a four-phase feeding program according to the Cobb 500 Broiler Performance and Nutrition Supplement (Cobb-Vantress, 2022) (Table 2, Table 3).

**Table 1:** Description of dietary treatments according to sex and nutritional programs in a 2 × 4 factorial arrangement.

Sex	Nutritional program	Rearing phases (days of age)				
		Phase 1	Phase 2	Phase 3	Phase 4	Phase 5
Male	BT 2024^1^	01 to 08	08 to 17	17 to 27	27 to 35	35 to 42
Male	BT 2017^2^	01 to 08	08 to 21	21 to 33	33 to 42	
Male	BT 1983^3^	01 to 27	27 to 42			
Male	Cobb 2022^4^	01 to 12	12 to 27	27 to 39	39 to 42	
Female	BT 2024^5^	01 to 08	08 to 17	17 to 27	27 to 35	35 to 42
Female	BT 2017^6^	01 to 08	08 to 21	21 to 33	33 to 42	
Female	BT 1983^3^	01 to 27	27 to 42			
Female	Cobb 2022^4^	01 to 12	12 to 27	27 to 39	39 to 42	

1 Recommendations according to Hannas et al. (2024) for high-performance male broilers.

2 Recommendations according to Rostagno et al. (2017) for standard-high performance male broilers.

3 Recommendations according to Rostagno et al. (1983) for as-hatched broilers.

4 Recommendations according to Cobb-Vantress (2022) for as-hatched broilers.

5 Recommendations according to Hannas et al. (2024) for high-performance female broilers.

6 Recommendations according to Rostagno et al. (2017) for standard-high performance female broilers.

**Table 2:** Composition of experimental diets according to nutritional programs for male broilers.

	BT 2024					BT 2017				BT 1983		Cobb 2022			
	1-8	8-17	17-27	27-35	35-42	1-8	8-21	21-33	33-42	1-27	27-42	1-12	12-27	27-39	39-42
ME, kcal/kg	2950	3000	3050	3100	3150	3000	3100	3200	3250	3000	3100	2900	2950	3050	3100
Lys/ME, %/Mcal	0.464	0.443	0.413	0.387	0.371	0.453	0.422	0.388	0.329	0.337	0.294	0.434	0.393	0.348	0.310
CP, %	25.52	24.94	23.06	22.09	21.24	25.47	24.42	22.62	19.60	20.79	19.13	23.64	21.87	20.07	18.27
SID Lys, %	1.37	1.33	1.26	1.20	1.17	1.36	1.31	1.24	1.07	1.01	0.91	1.26	1.16	1.06	0.96
SID Met, %	0.66	0.64	0.66	0.63	0.62	0.67	0.64	0.65	0.55	0.45	0.41	0.63	0.58	0.54	0.48
SID Met + Cys, %	1.00	0.97	0.97	0.93	0.91	1.01	0.97	0.95	0.82	0.74	0.68	0.94	0.88	0.82	0.74
SID Thr, %	0.90	0.88	0.83	0.79	0.77	0.90	0.86	0.82	0.70	0.71	0.66	0.86	0.78	0.70	0.62
SID Trp, %	0.30	0.29	0.27	0.25	0.24	0.30	0.29	0.26	0.22	0.24	0.22	0.27	0.25	0.23	0.20
SID Val, %	1.07	1.04	0.97	0.93	0.90	1.06	1.02	0.95	0.82	0.87	0.80	0.98	0.91	0.83	0.75
Ca, %	1.17	1.08	0.77	0.68	0.64	1.01	0.91	0.88	0.66	1.00	0.93	0.96	0.80	0.74	0.72
TP, %	0.82	0.77	0.62	0.57	0.54	0.74	0.68	0.55	0.54	0.74	0.69	0.83	0.65	0.60	0.59
AvP, %	0.56	0.51	0.37	0.32	0.30	0.48	0.43	0.30	0.31	0.50	0.47	0.58	0.40	0.37	0.36
Na, %	0.22	0.22	0.21	0.20	0.19	0.23	0.22	0.21	0.20	0.16	0.16	0.16	0.16	0.16	0.16
Cl, %	0.38	0.37	0.36	0.35	0.34	0.38	0.38	0.36	0.35	0.29	0.29	0.29	0.29	0.29	0.30
K, %	1.00	0.97	0.90	0.86	0.83	1.00	0.95	0.88	0.76	0.82	0.76	0.92	0.85	0.79	0.72

Abbreviations: ME, metabolizable energy; Lys/ME, lysine to metabolizable energy ratio; CP, crude protein; SID, standardized ileal digestible; Lys, lysine; Met, methionine; Cys, cystine; Thr, threonine; Trp, tryptophan; Val, valine; Ca, calcium; TP, total phosphorus; AvP, available phosphorus; Na, sodium; Cl, chloride; K, potassium.

**Table 3:** Composition of experimental diets according to nutritional programs for female broilers.

	BT 2024					BT 2017				BT 1983		Cobb 2022			
	1-8	8-17	17-27	27-35	35-42	1-8	8-21	21-33	33-42	1-27	27-42	1-12	12-27	27-39	39-42
ME, kcal/kg	2950	3000	3050	3100	3150	3000	3100	3200	3250	3000	3100	2900	2950	3050	3100
Lys/ME, %/Mcal	0.430	0.420	0.400	0.384	0.368	0.450	0.413	0.359	0.308	0.337	0.294	0.434	0.393	0.348	0.310
CP, %	23.71	23.62	22.41	21.80	21.06	25.17	23.94	21.07	18.38	20.79	19.13	23.64	21.87	20.07	18.27
SID Lys, %	1.27	1.26	1.22	1.19	1.16	1.35	1.28	1.15	1.00	1.01	0.91	1.26	1.16	1.06	0.96
SID Met, %	0.61	0.60	0.64	0.62	0.62	0.66	0.63	0.60	0.51	0.45	0.41	0.63	0.58	0.54	0.48
SID Met + Cys, %	0.92	0.92	0.94	0.91	0.91	1.00	0.95	0.88	0.77	0.74	0.68	0.94	0.88	0.82	0.74
SID Thr, %	0.84	0.83	0.81	0.78	0.77	0.89	0.84	0.76	0.66	0.71	0.66	0.86	0.78	0.70	0.62
SID Trp, %	0.28	0.28	0.26	0.25	0.24	0.30	0.28	0.24	0.20	0.24	0.22	0.27	0.25	0.23	0.20
SID Val, %	0.99	0.99	0.94	0.91	0.89	1.05	1.00	0.88	0.77	0.87	0.80	0.98	0.91	0.83	0.75
Ca, %	1.10	1.02	0.75	0.66	0.62	1.03	0.91	0.76	0.61	1.00	0.93	0.96	0.80	0.74	0.72
TP, %	0.78	0.74	0.60	0.56	0.54	0.75	0.68	0.59	0.51	0.74	0.69	0.83	0.65	0.60	0.59
AvP, %	0.53	0.49	0.35	0.31	0.30	0.49	0.43	0.35	0.28	0.50	0.47	0.58	0.40	0.37	0.36
Na, %	0.21	0.21	0.20	0.19	0.18	0.22	0.21	0.20	0.19	0.16	0.16	0.16	0.16	0.16	0.16
Cl, %	0.36	0.35	0.34	0.34	0.33	0.37	0.36	0.35	0.34	0.29	0.29	0.29	0.29	0.29	0.30
K, %	0.93	0.92	0.87	0.85	0.82	0.98	0.94	0.82	0.72	0.82	0.76	0.92	0.85	0.79	0.72

Abbreviations: ME, metabolizable energy; Lys/ME, lysine to metabolizable energy ratio; CP, crude protein; SID, standardized ileal digestible; Lys, lysine; Met, methionine; Cys, cystine; Thr, threonine; Trp, tryptophan; Val, valine; Ca, calcium; TP, total phosphorus; AvP, available phosphorus; Na, sodium; Cl, chloride; K, potassium.

The diets were based on corn, soybean meal, and soybean oil, with crystalline AA supplementation to meet the nutritional recommendations of each program and feed in mashed form. The crude protein and total amino acid contents of corn and soybean meal were analyzed by calibrated and validated near infrared spectroscopy (Lemme et al., 2020) (NIRS) before manufacturing feed and digestibility coefficients of the ingredients were considered according to Calderano et al. (2024). Dietary ME levels were adjusted to provide dLys:ME ratios in accordance with each nutritional program, as well as calcium and available phosphorus levels. In all phases, microminerals and vitamins were supplemented at equivalent levels across treatments.

### Growth performance

At the beginning, during the phase changes according to each nutritional plan and at the end of the experimental period, body weight, feed offered, and feed refusals were recorded to calculate average daily gain (**ADG**, kg/bird) and average daily feed intake (**ADFI**, kg/bird). ADFI was corrected for mortality according to Sakomura and Rostagno (2016). Feed conversion ratio (**FCR**, kg/kg) was calculated as ADFI divided by ADG.

### Body composition

At 27 and 42 days of age, one bird per replicate was selected (n = 80 birds per phase) based on the average weight of the experimental unit. The selected birds were fasted for 12 h and euthanized for analysis of body composition at the Laboratory of Body Composition and Densitometry (Federal University of Viçosa). Whole-body composition was assessed using dual-energy X-ray absorptiometry (**DXA**) with a Lunar Prodigy Advance scanner (GE Healthcare, Madison, WI, USA), equipped with enCORE 2011 software version 13.60.033. Before scanning, a quality assurance procedure was conducted using a phantom standard to ensure accurate calibration of the equipment. Birds were positioned dorsally on the scanning platform, with wings extended laterally and legs aligned to minimize anatomical superimposition. Scans were performed from cranial to caudal. A rectangular region of interest (**ROI**) encompassing the entire bird was manually defined for each scan. DXA provided raw outputs for soft lean tissue mass (**SLT**, g), fat tissue mass (**FAT**, g), and bone mineral content (**BMC**, g). The sum of these values was used to estimate total body weight (**BW**, g). These raw measurements were then converted into BW, SLT, protein (**PRO**, g), water (**WT**, g), fat (**FAT**), and ash (**ASH**, g) using prediction equations (Eqs. 1-6) validated for broilers by Aguiar et al. (2024).

BW (g) = 16.267 + 1.079 × BW (raw data); R² = 0.999 (1)

SLT (g) = − 31.807 + 1.157 × SLT (raw data); R² = 0.997 (2)

PRO (g) = − 8.871 + 0.217 × SLT (raw data); R² = 0.989 (3)

WT (g) = − 22.94 + 0.94 × SLT (raw data); R² = 0.997 (4)

FAT (g) = 11.817 + 0.756 × FAT (raw data); R² = 0.981 (5)

ASH (g) = 7.139 + 1.578 × BMC (raw data); R² = 0.956 (6)

### Carcass traits

At 42 days of age, one bird per replicate was selected (n = 80 birds) based on the average weight of the experimental unit. The selected birds were fasted for 12 h, weighed to obtain the slaughter weight (**SW**, kg), and euthanized by electronarcosis followed by bleeding. The carcass, eviscerated and with feet removed at the hock joint was weighed to obtain hot carcass weight (**HCW**, kg) and cold carcass weight (**CCW**, kg). Subsequently, the cuts were also weighed to obtain breast weight (**BTW**, kg), thigh weight (**THW**, kg), and drumstick weight (**DRW**, kg). The hot carcass yield (**HCY**, %) and cold carcass yield (**CCY**, %) were calculated as their weights divided by SW. The carcass yield was determined according to the main commercial part of breast meat and the leg, and the values of breast yield (**BTY**, %), thigh yield (**THY**, %), and drumstick yield (**DRY**, %) were expressed as percentage of carcass weight (Cömert et al. 2016).

### Statistical analysis

The pen was considered the experimental unit for ADG, ADFI, and FCR, whereas the individual bird was considered the experimental unit for body composition and carcass traits. All statistical procedures were performed using R (version 4.2.1; R Core Team, 2022). Data were analyzed using of variance (ANOVA) at a 95 % confidence level in a 2 × 4 factorial arrangements including the main effects of sex, nutritional programs, and their interaction (Eq. 7). When significant effects were identified, means were separated using the Student-Newman-Keuls (**SNK**) test at P < 0.05.

(7) Yijk=μ+Si+Nj+(T×E)ij+eijk,

where Yijk is the response variable, Si is the effect of sex (i = 1,2), Nj is the effect of nutritional programs (j = 1,2,3,4), (S x N)ij is the interaction effect between sex and nutritional programs, and eijk is the random error term.

### Economic analysis

The economic analysis considered four variables: feed cost per bird, feed cost per kilogram of ADG, gross revenue, and net revenue per bird, expressed in USD/bird. Feed cost per bird was calculated as the ADFI multiplied by the corresponding diet price. From this value, feed cost per kilogram of ADG was obtained by dividing feed cost per bird by the ADG of the respective treatment. Gross revenue was determined by multiplying ADG by the market price of live broilers, and net revenue was calculated as the difference between gross revenue and feed cost per bird. The prices of inputs were obtained in the market surveys in the Viçosa region and for corn and soybean meal from the company Pif Paf Alimentos (Visconde do Rio Branco, MG, Brazil). Live broiler prices were estimated at 0.89 USD based on data provided by the Institute of Agricultural Economics (IEA), the Rural Economy Division of the Secretariat of Agriculture and Supply of Paraná (DERAL/DEB-SEAB/PR), and Santa Catarina's Agricultural Research and Rural Extension Company (EPAGRI/CEPA). Prices per kilogram were expressed in dollars, quotation used between January and February 2024: corn 0.27; soybean meal 0.46; soybean oil 1.34; dicalcium phosphate 0.59; limestone 0.04; salt 0.12; DL-methionine 1.56; L-lysine HCl 1.37; L-threonine 2.21; L-valine 4.60; mineral supplement 1.17; vitamin supplement 1.56; choline chloride 2.37; anticoccidial 1.82, additive growth enhancer 15.66 and antioxidant 2.46. The exchange rate (BRL to USD) was calculated using the average quotations for the same period (1 USD = 5.11 BRL).

## Results

There was a sex × nutritional program interaction for ADFI (P = 0.030), ADG (P = 0.012), and FCR (P < 0.001) in broilers from 01 to 42 d of age (Table 4). Within males, ADFI was highest in BT 1983 and lowest in BT 2024, whereas ADG was greatest in BT 2017 and lowest in BT 1983 and Cobb 2022. BT 2024 showed higher ADG than BT 1983 and Cobb 2022. FCR was highest in BT 1983, intermediate in BT 2017 and Cobb 2022, and lowest in BT 2024. Within females, ADFI was also highest in BT 1983 and lowest in BT 2024. ADG was greatest in BT 2017 and lowest in BT 1983. FCR showed the same pattern as in males, with the highest values in BT 1983, intermediate values in BT 2017 and Cobb 2022, and the lowest in BT 2024. Within nutritional programs, males showed higher ADFI and ADG than females in all programs, and lower FCR in BT 2024 and BT 1983.

**Table 4:** Interactive and main effects of sex and nutritional programs on growth performance of broilers from 01 to 42 days.

Treatments	ADFI (kg/bird)	ADG (kg/bird)	FCR
	Interactive effect of sex and nutritional programs		
Male × BT 2024	5.117 ^D,^^a^	3.410 B^,^^a^	1.501^C^^,^^b^
Male × BT 2017	5.920 B^,^^a^	3.536^A^^,^^a^	1.674 B
Male × BT 1983	6.408^A^^,^^a^	3.156 C^,^^a^	2.032^A^^,^^b^
Male × Cobb 2022	5.406 C^,^^a^	3.221 C^,^^a^	1.684 B
Female × BT 2024	4.638^D^^,^^b^	2.928 A^B,^^b^	1.585 C^,^^a^
Female × BT 2017	5.153^B^^,^^b^	2.997^A^^,^^b^	1.716 B
Female × BT 1983	5.884^A^^,^^b^	2.745 ^C,^^b^	2.164 ^A,^^a^
Female × Cobb 2022	4.810 C^,^^b^	2.847 B^,^^b^	1.677 B
	**Main effect of sex**		
Male	5.715	3.331	1.724
Female	5.098	2.877	1.789
	**Main effect of the nutritional program**		
BT 2024	4.887	3.169	1.543
BT 2017	5.558	3.280	1.693
BT 1983	6.159	2.950	2.098
Cobb 2022	5.092	3.044	1.680
	**P-value**		
Sex	<0.001	<0.001	<0.001
Nutritional program	<0.001	<0.001	<0.001
Sex × nutritional program	0.030	0.012	<0.001
SEM	0.0690	0.0312	0.0252

A-D Uppercase letters represent differences between nutritional programs (BT 2024, BT 2017, BT 1983, and Coob 2022) within each sex (male or female) in the same column (SNK, P < 0.05).

a-b Lowercase letters represent differences between sex (male and female) within each nutritional program (BT 2024, BT 2017, BT 1983, or Cobb 2022) in the same column (SNK, P < 0.05). SEM = Standard Error of the Mean (n = 10 for treatment). Abbreviations: ADFI, average daily feed intake; ADG, average daily gain; FCR, feed conversion ratio.

The sex × nutritional program interaction affected BW, SLT, PRO, and WT (P < 0.001) in broilers at 27 d of age (Table 5). Within males, BW was highest in BT 2017 and lowest in BT 1983. SLT, PRO, and WT were greatest in BT 2017 and BT 2024, intermediate in Cobb 2022, and lowest in BT 1983. Within females, BW in BT 1983 was lower than in BT 2024, BT 2017, and Cobb 2022. For SLT, PRO, and WT, the greatest values were observed in BT 2017, intermediate in BT 2024 and Cobb 2022, and the lowest in BT 1983. Within nutritional programs, males showed higher BW, SLT, PRO, and WT than females. There was no interaction for FAT (P = 0.496) and ASH (P = 0.465). However, FAT was affected by nutritional program (P < 0.001), with higher values in BT 2017 and BT 1983 than in BT 2024 and Cobb 2022. ASH was affected by sex (P < 0.001), with higher values in males than in females.

**Table 5:** Interactive and main effects of sex and nutritional programs on carcass composition of broilers slaughtered at 27 d of age.

Treatments	BW (g)	SLT (g)	PRO (g)	WT (g)	FAT (g)	ASH (g)
	Interactive effect of sex and nutritional programs					
Male × BT 2024	1886 B^,^^a^	1750 A^,^^a^	325 A^,^^a^	1425 A^,^^a^	106	48
Male × BT 2017	1960 A^,^^a^	1778 A^,^^a^	330 A^,^^a^	1448 A^,^^a^	125	47
Male × BT 1983	1626^D^^,^^a^	1453^C^^,^^a^	269 ^C,^^a^	1183 ^C,^^a^	120	46
Male × Cobb 2022	1713 ^C,^^a^	1574 B^,^^a^	291 B^,^^a^	1281 B^,^^a^	103	47
Female × BT 2024	1589 A^,^^b^	1453 AB^,^^b^	269 AB^,^^b^	1183 AB^,^^b^	113	42
Female × BT 2017	1638 A^,^^b^	1503 A^,^^b^	278 A^,^^b^	1225 A^,^^b^	116	42
Female × BT 1983	1516 B^,^^b^	1351 ^C,^^b^	250 ^C,^^b^	1101 ^C,^^b^	124	39
Female × Cobb 2022	1580 A^,^^b^	1430 B^,^^b^	265 B^,^^b^	1165 B^,^^b^	101	41
	**Main effect of sex**					
Male	1797	1640	304	1335	113	46 A
Female	1579	1432	265	1166	113	41 B
	**Main effect of the nutritional program**					
BT 2024	1745	1609	298	1310	109 B	44
BT 2017	1808	1641	304	1336	120 A	44
BT 1983	1571	1399	259	1139	122 A	43
Cobb 2022	1646	1502	278	1223	102 B	42
	**P-value**					
Sex	<0.001	<0.001	<0.001	<0.001	0.979	<0.001
Nutritional program	<0.001	<0.001	<0.001	<0.001	<0.001	0.097
Sex × Nutritional program	<0.001	<0.001	<0.001	<0.001	0.496	0.465
SEM	18.390	18.154	3.400	14.749	2.009	0.448

A-D Uppercase letters represent differences between nutritional programs (BT 2024, BT 2017, BT 1983, and Cobb 2022) within each sex (male or female) in the same column (SNK, P < 0.05).

a-b Lowercase letters represent differences between sex (male and female) within each nutritional program (BT 2024, BT 2017, BT 1983, or Cobb 2022) in the same column (SNK, P < 0.05). SEM: Standard Error of the Mean (n = 10 for treatment). Abbreviations: BW, total body weight; SLT, soft lean tissue; PRO, protein; WT, water; FAT, fat; ASH, ash. Whole-body composition was assessed using dual-energy X-ray absorptiometry (DXA).

There was no interaction between sex and nutritional programs for BW (P = 0.197), SLT (P = 0.396), PRO (P = 0.396), WT (P = 0.317), FAT (P = 0.691), and ASH (P = 0.626) in broilers at 42 d of age (Table 6). A main effect of sex was observed for BW, SLT, PRO, WT, and ASH (P < 0.001), with higher values in males than females. A main effect of the nutritional program was observed for BW, SLT, PRO, WT, and FAT (P < 0.001). BW was lower in BT 1983 than in BT 2024, BT 2017, and Cobb 2022. SLT, PRO, and WT were greatest in BT 2024 and BT 2017, intermediate in Cobb 2022, and lowest in BT 1983. FAT was highest in BT 2017, intermediate in BT 1983 and Cobb 2022, and lowest in BT 2024.

**Table 6:** Interactive and main effects of sex and nutritional programs on carcass composition of broilers slaughtered at 42 d of age.

Treatments	BW (g)	SLT (g)	PRO (g)	WT (g)	FAT (g)	ASH (g)
	Interactive effect of sex and nutritional programs					
Male × BT 2024	3676	3407	635	2771	244	70
Male × BT 2017	3818	3446	642	2802	293	69
Male × BT 1983	3039	2994	557	2436	271	72
Male × Cobb 2022	3572	3249	605	2642	239	73
Female × BT 2024	3174	2825	526	2298	239	57
Female × BT 2017	3195	2817	524	2292	316	59
Female × BT 1983	2904	2514	468	2046	285	58
Female × Cobb 2022	3062	2713	505	2261	264	61
	**Main effect of sex**					
Male	3527^A^	3274 A	610 A	2663 A	262	71 A
Female	3083^B^	2720^B^	506^B^	2227^B^	274	59^B^
	**Main effect of the nutritional program**					
BT 2024	3425^A^	3116 A	580 A	2535 A	241 C	64
BT 2017	3541^A^	3148 A	586 A	2560 A	304 A	64
BT 1983	2975 B	2767^C^	515 C	2251 C	278 A^B^	64
Cobb 2022	3317^A^	2995^B^	558^B^	2451^B^	252^B^^C^	67
	**P-value**					
Sex	<0.001	<0.001	<0.001	<0.001	0.177	<0.001
Nutritional program	<0.001	<0.001	<0.001	<0.001	<0.001	0.227
Sex × Nutritional program	0.197	0.396	0.396	0.317	0.691	0.626
SEM	53.61	39.334	7.367	31.763	5.626	0.912

A-D Uppercase letters represent differences between nutritional programs (BT 2024, BT 2017, BT 1983, and Cobb 2022) within each sex (male or female) in the same column (SNK, P < 0.05). ^a-b^Lowercase letters represent differences between sex (male and female) within each nutritional program (BT 2024, BT 2017, BT 1983, or Cobb 2022) in the same column (SNK, P < 0.05). SEM: Standard Error of the Mean (n = 10 for treatment). Abbreviations: BW, total body weight; SLT, soft lean tissue; PRO, protein; WT, water; FAT, fat; ASH, ash. Whole-body composition was assessed using dual-energy X-ray absorptiometry (DXA).

The sex × nutritional program interaction affected SW (P < 0.001) and THW (P = 0.012) in broilers at 42 d of age (Table 7). Within males, SW was higher in BT 2024 and BT 2017, intermediate in Cobb 2022, and lower in BT 1983. Within females, SW in BT 1983 was lower than in BT 2024, BT 2017, and Cobb 2022. Within nutritional programs, males showed higher SW and THW than females. There was no interaction for HCW (P = 0.091), CCW (P = 0.674), BTW (P = 0.125), and DRW (P = 0.396). However, main effects were observed for sex and nutritional program (P < 0.001). Males showed higher HCW, CCW, BTW, and DRW than females. Among nutritional programs, HCW, CCW, and BTW were greatest in BT 2024 and BT 2017, intermediate in Cobb 2022, and lowest in BT 1983. DRW was higher in BT 2017 than in BT 2024, BT 1983, and Cobb 2022.

**Table 7:** Interactive and main effects of sex and nutritional programs on carcass weight of broilers slaughtered at 42 d of age.

Treatments	SW (kg)	HCW (kg)	CCW (kg)	BTW (kg)	THW (kg)	DRW (kg)
	Interactive effect of sex and nutritional programs					
Male × BT 2024	3.532 A^,^^a^	2.778	2.838	1.091	0.363 A^,^^a^	0.387
Male × BT 2017	3.528 A^,^^a^	2.811	2.894	1.064	0.369 A^,^^a^	0.422
Male × BT 1983	3.175 ^C,^^a^	2.553	2.642	0.908	0.348 A^,^^a^	0.385
Male × Cobb 2022	3.293 B^,^^a^	2.612	2.718	0.989	0.346 A^,^^a^	0.390
Female × BT 2024	2.936 A^,^^b^	2.373	2.480	0.921	0.304 A^,^^b^	0.336
Female × BT 2017	2.975 A^,^^b^	2.410	2.479	0.955	0.280 AB^,^^b^	0.338
Female × BT 1983	2.741 B^,^^b^	2.208	2.296	0.859	0.258 B^,^^b^	0.310
Female × Cobb 2022	2.921 A^,^^b^	2.344	2.392	0.890	0.301 A^,^^b^	0.332
	**Main effect of sex**					
Male	3.380	2.691 A	2.772 A	1.011 A	0.356	0.397 A
Female	2.887	2.331 B	2.410 B	0.905 B	0.286	0.329 B
	**Main effect of the nutritional program**					
BT 2024	3.234	2.565 A	2.650 A	1.001 A	0.330	0.362 B
BT 2017	3.282	2.621^A^	2.698 A	1.013 A	0.325	0.383 A
BT 1983	2.958	2.385^C^	2.469^C^	0.884^C^	0.303	0.347 B
Cobb 2022	3.107	2.478^B^	2.555 B	0.942 B	0.323	0.347 B
	**P-value**					
Sex	<0.001	<0.001	<0.001	<0.001	<0.001	<0.001
Nutritional program	<0.001	<0.001	<0.001	<0.001	<0.001	<0.001
Sex × Nutritional program	<0.001	0.091	0.674	0.125	0.012	0.396
SEM	0.0340	0.0254	0.0262	0.0122	0.0051	0.0054

A-D Uppercase letters represent differences between nutritional programs (BT 2024, BT 2017, BT 1983, and Cobb 2022) within each sex (male or female) in the same column (SNK, P < 0.05).

a-b Lowercase letters represent differences between sex (male and female) within each nutritional program (BT 2024, BT 2017, BT 1983, or Cobb 2022) in the same column (SNK, P < 0.05). SEM: Standard Error of the Mean (n = 10 for treatment). Abbreviations: SW, slaughter weight; HCW, hot carcass weight; CCW, cold carcass weight; BTW, breast weight; THW, thighs weight; DRW, drumstick weight.

The sex × nutritional program interaction affected THY (P = 0.012) in broilers at 42 d of age (Table 8). Within females, THY was higher in BT 2024 and Cobb 2022 than in BT 2017 and BT 1983. Within nutritional programs, males showed higher THY than females in BT 2017 and BT 1983. There was no interaction for BTY (P = 0.309) and DRY (P = 0.420). However, a main effect of sex was observed for BTY (P = 0.034) and DRY (P < 0.001), with lower BTY and higher DRY in males than females. HCY and CCY were not affected by treatments (P > 0.05).

**Table 8:** Interactive and main effects of sex and nutritional programs on carcass yield of broilers slaughtered at 42 d of age.

Treatments	HCY (%)	CCY (%)	BTY (%)	THY (%)	DRY (%)
	**Interactive effect of sex and nutritional programs**				
Male × BT 2024	79.34	80.88	37.76	12.69	13.80
Male × BT 2017	80.02	82.06	36.78	12.58^a^	14.59
Male × BT 1983	79.78	83.29	35.44	13.19^a^	14.66
Male × Cobb 2022	79.16	81.76	36.38	12.75	14.36
Female × BT 2024	80.01	83.58	37.13	12.30^A^	13.57
Female × BT 2017	80.33	82.85	38.51	11.33^B^^,^^b^	13.66
Female × BT 1983	79.82	82.82	37.39	11.28^B^^,^^b^	13.46
Female × Cobb 2022	80.20	83.20	37.98	12.56^A^	13.57
	**Main effect of sex**				
					
Male	80.08	83.12	36.59^B^	12.80	14.35^A^
Female	79.56	82.03	37.73^A^	11.88	13.57^B^
	**Main effect of the nutritional program**				
BT 2024	79.69	82.30	37.43	12.48	13.69
BT 2017	80.17	82.43	37.60	11.99	14.15
BT 1983	79.80	83.07	36.47	12.23	14.06
Cobb 2022	79.68	82.44	37.18	12.65	13.99
	**P-value**				
Sex	0.181	0.069	0.034	<0.001	<0.001
Nutritional program	0.811	0.809	0.479	0.120	0.431
Sex × Nutritional program	0.814	0.314	0.309	0.012	0.420
SEM	0.192	0.302	0.277	0.121	0.113

A-D Uppercase letters represent differences between nutritional programs (BT 2024, BT 2017, BT 1983, and Cobb 2022) within each sex (male or female) in the same column (SNK, P < 0.05).

a-b Lowercase letters represent differences between sex (male and female) within each nutritional program (BT 2024, BT 2017, BT 1983, or Cobb 2022) in the same column (SNK, P < 0.05). SEM: Standard Error of the Mean (n = 10 for treatment). Abbreviations: HCY, hot carcass yield; CCY, cold carcass yield; BTY, breast yield; THY, thighs yield; DRY, drumstick yield.

The economic analysis showed that, for both males and females, feed cost per bird was lowest in Cobb 2022. Gross revenue was highest in BT 2017, whereas feed cost per kilogram of ADG was lowest in BT 2024, which consequently showed the highest net revenue (Table 9).

**Table 9:** Economic (USD) analysis of feed cost, gross revenue, and net revenue of male and female broilers fed different nutritional programs.

	BT 2024	BT 2017	BT 1983	Cobb 2022
*Males*				
Feed cost per bird	1.95	2.37	2.27	1.96
Gross revenue	3.02	3.13	2.79	2.85
Feed cost per kg ADG	0.57	0.67	0.72	0.61
Net revenue	1.07	0.77	0.52	0.89
*Females*				
Feed cost per bird	1.74	1.98	2.11	1.73
Gross revenue	2.59	2.65	2.43	2.54
Feed cost per kg ADG	0.59	0.77	0.77	0.60
Net revenue $ per bird	0.85	0.66	0.32	0.81

Dollar quotation: 1 USD = 5.11 BRL.

## Discussion

In the present study, the factorial arrangement proved effective in identifying the influence of sex and nutritional programs on broiler chicken production.

In recent years, broiler nutrition has progressed from diets based on common requirements for both sexes to sex-specific recommendations, incorporating concepts such as ideal protein and nitrogen-corrected ME, along with continuous updates of nutrient requirement tables (Elwinger et al., 2016). As a result, nutrition has supported the productive potential of modern genotypes and has become a key driver of efficiency in broiler production.

Comparing the last 41 years of advancements in broiler nutrition, through the nutritional programs BT 1983 and BT 2024, substantial improvements in growth performance were observed in both sexes in the updated program. On average, ADFI was reduced by about 20 %, while ADG increased by around 7 %, resulting in an improvement of approximately 26 % in FCR in both sexes. These gains were reflected in carcass traits, with an increase of about 20 % in SLT, indicating greater protein deposition. Consequently, carcass weight increased by 7 %, with emphasis on breast yield, which showed a 13 % increase.

The BT 1983 nutritional program was divided into two phases and was formulated for both males and females (as hatched), whereas the BT 2024 was divided into five phases and was formulated for males and females separately. Studies report that multiphase feeding in broilers provides better adjustment of nutrient supply to the birds’ requirements, since fewer feeding phases based on average requirements tend to either overestimate or underestimate nutrient levels during most of the growth period. By allowing more frequent adjustments, multiphase programs match more closely the actual nutritional needs, resulting in improved growth performance and economic efficiency (Hauschild et al., 2015; Tikate et al., 2021). Additionally, it has been reported that males and females have different nutritional requirements. Male broilers have higher nutritional requirements compared to females, which highlights the potential benefits of separate-sex diets, allowing a more precise feed formulation (England et al., 2022). Therefore, the superior performance obtained with BT 2024 may be attributed to its more precise formulation, meeting the requirements of each sex in each phase.

Another advancement from BT 1983 to BT 2024 is related to AA recommendations, especially Lys. In the BT 2024 program, dietary digestible Lys ranged from 1.37 to 1.17 % for males and 1.27 to 1.16 % for females, whereas in BT 1983 it ranged from 1.01 to 0.91 %. Lys is the second limiting AA in diets based on corn and soybean meal for broilers and is directly associated with muscle growth and the efficient utilization of other AA (An et al., 2023). Because Lys is used exclusively for protein deposition and maintenance, it serves as a reliable indicator for estimating nutritional requirements. According to Nogueira et al. (2021), broilers require approximately 95 mg of digestible Lys to deposit 1 g of body protein, a value consistent between males (94.9 mg) and females (92.9 mg). Therefore, the adoption of the outdated program, with lower recommendations for Lys, may have limited the potential growth of modern strains.

Although it is crucial to update the AA recommendations, considering the absolute intake of AA and its relative content to ME may be more important for tissue synthesis and optimization of performance (Barekatain et al., 2021). Studies have reported that the dLys:ME ratio is crucial to optimize growth performance and carcass traits in broiler chickens. This is because an excess supply of dietary energy primarily promotes fat deposition, impairing feed efficiency and reducing meat yield, whereas increasing AA density, particularly Lys, supports protein accretion and muscle growth. Therefore, increasing the dLys:ME ratio helps to maintain ADG and improve FCR, ensuring that nutrient intake favors lean tissue deposition rather than fat accumulation (Sharma et al., 2018; Maharjan et al., 2020; Maharjan et al., 2021; Mansilla et al., 2022; Toghyani et al., 2024). Barekatain et al. (2021) reported that birds fed diets containing 0.396 and 0.437 % per Mcal dLys:ME consumed a similar amount of feed but more than birds fed 0.355 % per Mcal dLys:ME. As a consequence, the FCR decreased as dLys:ME ratios increased from 0.355 to 0.437 % per Mcal. Similarly, Toghyani et al. (2024), using broken-line models, determined optimal dLys:ME ratios across different feeding phases in broiler chickens. In the starter phase (0-10 d), the dLys:ME ratios that optimized ADG and FCR were estimated at 0.466 and 0.482 % per Mcal, respectively. During the grower phase (10-24 d), the optimal dLys:ME ratios were 0.404 and 0.411 % per Mcal for ADG and FCR, respectively. In the finisher phase (24-35 d), the model could not be fitted for ADG, but the optimal dLys:ME ratio for FCR was estimated at 0.363 % per Mcal. Conversely, in the withdrawal phase (35-42 d), the model could not be fitted for FCR, but the optimal dLys:ME ratio for ADG was estimated at 0.345 % per Mcal. In the present study, the dLys:ME ranged from 0.464 to 0.371 % per Mcal for males and from 4.30 to 3.68 % per Mcal for females in BT 2024, whereas it ranged from 3.37 to 2.94 % per Mcal in BT 1983. This higher ratio between AA to energy density in the update program may help to understand the improvements in performance and carcass traits.

Beyond the comparison between BT 1983 and BT 2024, it is important to note that both BT 2017 and Cobb 2022 represented substantial advancements over BT 1983, with higher growth performance and carcass traits.

When comparing the recent advances of the last 7 years through the nutritional programs BT 2017 and BT 2024, improvements in broiler growth performance were also observed. In both males and females, ADFI decreased by 13.6 % and 10 %, respectively. Notably, there was a decrease in ADG by 3.6 % between BT 2017 and BT 2024 for males, whereas there were no differences for females. However, even with the lower ADG in the updated program, the FCR was improved by 10.3 % in males and by 7.6 % in females. In addition to the improvement in feed utilization observed with the BT 2024 program, the lower ADG recorded in males was not reflected in carcass traits, as no differences were found in carcass or breast weight. Furthermore, when evaluating body composition at 27 d of age, although BT 2017 showed higher estimated BW, SLT, PRO, and WT did not differ between BT 2017 and BT 2024, indicating that BT 2024 was more efficient in protein accretion, whereas BT 2017 resulted in greater deposition of non-carcass components, such as intestines. Additionally, the FAT content in the carcass was lower in BT 2024 at 27 and 42 days. These results indicate that the BT 2024 program was more efficient in feed utilization and protein deposition, reinforcing that the BT 2024 nutritional program favored protein rather than fat accretion.

Analyzing the main differences between the programs, BT 2017 was divided into four phases, whereas BT 2024 was divided into five phases. Increasing the number of phases in broiler feeding programs allows a more precise adjustment between nutrient supply and the physiological requirements of birds at each stage of growth, minimizing both the overestimation and underestimation of nutrient levels (Hauschild et al., 2015; Tikate et al., 2021). In systems with fewer phases, it is common that, for a large part of the cycle, birds receive nutrients in excess or in deficiency, which compromises the efficiency of ingredient utilization (Hauschild et al. 2015b; Moss et al. 2021). By adopting more phases, the fractionation of diets enables gradual and continuous adjustments, ensuring nutrient intake is closer to actual requirements, which improves feed utilization, enhances feed efficiency, and results in improved FCR (Hauschild et al., 2015). This alignment between supply and requirements reduces the restriction periods followed by compensatory growth, a mechanism that rarely generates an increase in meat yield (Hauschild et al., 2015). Therefore, updated nutritional programs with a greater number of phases contribute to optimizing productive performance by improving nutrient utilization. Additionally, the dLys:ME ratios in BT 2024 were slightly higher than those in BT 2017 across the rearing phases. This higher proportion of AA relative to energy density may explain the greater protein rather than fat accretion observed in BT 2024.

As genetic progress has continually enhanced the growth potential, feed efficiency, and robustness of modern broilers, breeding companies provide updated nutritional recommendations to ensure that nutrient supply is aligned with the performance potential of the latest strains (Cobb-Vantress, 2022). Although the Cobb 2022 nutritional program has been recently updated, with modern concepts, it provides nutrient levels for small, medium, and large broilers without sex-specific recommendations. According to Hannas et al. (2024), the requirements for high-performance male broilers range from 0.220 to 2.486 g/day for SID Lys and 47.5 to 680.4 kcal/day for ME, whereas the requirements for high-performance female broilers range from 0.200 to 2.265 g/day for SID Lys and 46.6 to 623.9 kcal/day for ME. Given the differences in requirements between sexes, recommendations should be sex-specific to optimize performance. This was evident in our findings, as the programs that formulated diets specifically for males and females (BT 2024 and BT 2017) were superior to the Cobb 2022 recommendations, which did not consider sex separation.

Regarding the effects of sex, ample evidence in the literature indicates consistent differences between sexes, with males having heavier BW and higher ADFI than females (England et al., 2022). Our findings are in agreement, showing that male broilers exhibited superior performance to females, expressed as higher ADFI and ADG across all nutritional programs, and lower FCR in BT 2024 and BT 1983. Similarly, Freitas et al. (2025), analyzing 4,842 flocks using big data, reported that flocks composed of males showed higher ADG and lower FCR than female flocks. The differences between sexes cannot be attributed to a single factor but may involve differences in nutritional requirements and different hormone levels (Zerehdaran et al., 2005).

Because males are heavier than females at the same age, their carcass parts are also heavier (England et al., 2022). This was confirmed in our study, where body composition (SLT, PRO, WT, and ASH) and carcass and cuts weights were higher in males than in females at 27 and 42 d of age. Although males demonstrated higher carcass and cut weights, females yielded greater BTY and lower DRY than males, which corroborates previous reports (Young et al., 2001; Maynard et al., 2023).

From an economic perspective, the BT 2024 nutritional program resulted in greater profits compared to the other programs in both males and females. This is evident when analyzing net revenue, calculated as gross revenue minus feed cost per bird. Although BT 2017 demonstrated the highest gross revenue, as ADG was higher, particularly in males, the feed cost per bird was also the highest. As a consequence, the net revenue was lower than that of BT 2024 and Cobb 2022, and higher only than the outdated program BT 1983, for both males and females. In contrast, the most recent updated program BT 2024 provided lower gross revenue than BT 2017 due to lower ADG. However, the feed cost per bird was the lowest in males and the second lowest in females, which resulted in the highest net revenue for both sexes.

In conclusion, the progressive updates in broiler nutritional programs over the years have provided a more accurate nutrient supply, improved feed utilization, and enhanced carcass traits. Accordingly, the most recent update, BT 2024 nutritional recommendation with sex specific phase feeding, demonstrates superior performance and economic efficiency. These findings highlight the importance of continuously updating nutritional recommendations to meet the performance potential of modern strains and ensure sustainable and profitable production.

## Funding

This work was supported by the Brazilian funding agencies, the Program of Academic Excellence (PROEX) from the Coordenação de Aperfeiçoamento de Pessoal de Nível Superior (CAPES, process no. 88887.844747/2023-00), the Conselho Nacional de Desenvolvimento Científico e Tecnológico (CNPq), the Fundação de Amparo à Pesquisa do Estado de Minas Gerais (FAPEMIG), and the Instituto Nacional de Ciência e Tecnologia de Ciência Animal (INCT-CA).

## CRediT authorship contribution statement

**Filipe A. Monteiro:** Writing – original draft, Validation, Investigation, Data curation. **Carlos H. Oliveira:** Writing – review & editing, Writing – original draft, Visualization. **Maria R.S. Farias:** Validation, Investigation, Formal analysis, Data curation. **Lucimauro Fonseca:** Validation, Formal analysis, Data curation. **Bruno T. Ramos:** Validation, Investigation, Data curation. **Gabriel R. Braga:** Validation, Investigation, Formal analysis, Data curation. **Sarah M. Andrade:** Validation, Investigation, Data curation. **Arele A. Calderano:** Writing – review & editing, Visualization, Methodology, Conceptualization. **Horacio S. Rostagno:** Writing – review & editing, Visualization, Methodology, Conceptualization. **Melissa I. Hannas:** Writing – review & editing, Writing – original draft, Visualization, Validation, Supervision, Resources, Project administration, Methodology, Investigation, Funding acquisition, Data curation, Conceptualization.

## Disclosures

“The authors declare that they have no competing interest relative with this manuscript”

## Appendix Supplementary materials

Image, application 1
