# Supplementary material for: Updated nutritional program improves performance, carcass yield, and profitability in male and female broilers
Source: Poult Sci. 2025 Dec 13;105(2):106283. doi: 10.1016/j.psj.2025.106283 (PMC12799911; doi:10.1016/j.psj.2025.106283)
Supplement: Supplementary file 1 [file mmc1.docx]

**Supplementary Material 1.** Composition of diets from the 2024 Brazilian Tables program (BT 2024), formulated to meet the nutrient requirements of high-performance male and female broilers, according to Hannas et al. (2024).

| **Ingredients** | **High-Performance Male** | | | | |  | **High-Performance Female** | | | | |
| --- | --- | --- | --- | --- | --- | --- | --- | --- | --- | --- | --- |
|  | **1-8** | **8-17** | **17-27** | **27-35** | **35-42** |  | **1-8** | **8-17** | **17-27** | **27-35** | **35-42** |
|  |  |  |  |  |  |  |  |  |  |  |  |
| Corn, 7.88% | 45.160 | 46.522 | 53.705 | 56.340 | 58.368 |  | 51.272 | 51.045 | 55.972 | 59.054 | 60.366 |
| Soybean meal, 46% | 46.598 | 45.140 | 39.684 | 37.171 | 34.88 |  | 41.705 | 41.562 | 37.905 | 34.388 | 33.101 |
| Soybean oil | 3.305 | 3.755 | 2.995 | 3.250 | 3.635 |  | 2.315 | 3.010 | 2.615 | 3.505 | 3.930 |
| Dicalcium phosphate | 2.340 | 2.110 | 1.360 | 1.150 | 1.050 |  | 2.200 | 2.000 | 1.310 | 1.040 | 0.700 |
| Limestone | 1.120 | 1.040 | 0.770 | 0.690 | 0.650 |  | 1.080 | 1.000 | 0.750 | 0.620 | 0.540 |
| Salt | 0.525 | 0.510 | 0.490 | 0.470 | 0.455 |  | 0.500 | 0.485 | 0.460 | 0.430 | 0.430 |
| L-Lysine HCl | 0.089 | 0.093 | 0.156 | 0.164 | 0.196 |  | 0.107 | 0.107 | 0.164 | 0.199 | 0.203 |
| DL-Methionine | 0.328 | 0.318 | 0.353 | 0.333 | 0.341 |  | 0.293 | 0.292 | 0.339 | 0.338 | 0.327 |
| L-Threonine | 0.041 | 0.038 | 0.051 | 0.047 | 0.057 |  | 0.033 | 0.033 | 0.049 | 0.057 | 0.055 |
| L-Valine | - | - | 0.011 | 0.011 | 0.027 |  | - | - | 0.012 | 0.028 | 0.028 |
| Vitamin supplement^1^ | 0.145 | 0.136 | 0.120 | 0.100 | 0.090 |  | 0.145 | 0.136 | 0.120 | 0.090 | 0.082 |
| Mineral supplement^2^ | 0.145 | 0.136 | 0.120 | 0.100 | 0.090 |  | 0.145 | 0.136 | 0.120 | 0.090 | 0.082 |
| Choline chloride, 60% | 0.104 | 0.101 | 0.084 | 0.074 | 0.061 |  | 0.104 | 0.095 | 0.084 | 0.061 | 0.057 |
| Salinomicin, 12% | 0.036 | 0.036 | 0.036 | 0.036 | 0.036 |  | 0.036 | 0.036 | 0.036 | 0.036 | 0.036 |
| Antioxidant BHT^3^ | 0.050 | 0.050 | 0.050 | 0.050 | 0.050 |  | 0.050 | 0.050 | 0.050 | 0.050 | 0.050 |
| Avilamycin, 10% | 0.015 | 0.015 | 0.015 | 0.015 | 0.015 |  | 0.015 | 0.015 | 0.015 | 0.015 | 0.015 |

^1^Supplied per kg of vitamin supplement: Vitamin A 9,750 IU; Vitamin D₃ 2,470 IU; Vitamin E 36.6 IU; Vitamin B₁ 2.60 mg; Vitamin B₂ 6.50 mg; Vitamin B₆ 3.64 mg; Vitamin B₁₂ 0.015 mg; Pantothenic acid 13.0 mg; Biotin 0.091 mg; Vitamin K₃ 1.95 mg; Folic acid 0.91 mg; Nicotinic acid 39.0 mg. ^2^Supplied per kg of mineral supplement: iron 45.51 g; copper 9,480 mg; iodine 965 mg; manganese 66.47 g; selenium 287 mg; zinc 62.32 g. ^3^Butylated hydroxytoluene.

**Supplementary Material 2.** Composition of diets from the 2017 Brazilian Tables program (BT 2017), formulated to meet the nutrient requirements of standard-high performance male and female broilers, according to Rostagno et al. (2017).

| **Ingredients** | **Standard-High Performance Male** | | | |  | **Standard-High Performance Female** | | | |
| --- | --- | --- | --- | --- | --- | --- | --- | --- | --- |
|  | **1-8** | **8-21** | **21-33** | **33-42** |  | **1-8** | **8-21** | **21-33** | **33-42** |
|  |  |  |  |  |  |  |  |  |  |
| Corn, 7.88% | 45.275 | 47.087 | 51.667 | 60.844 |  | 46.143 | 48.604 | 56.683 | 65.103 |
| Soybean meal, 46% | 46.469 | 43.921 | 39.094 | 31.086 |  | 45.666 | 42.636 | 34.924 | 27.744 |
| Soybean oil | 3.855 | 5.005 | 5.645 | 4.915 |  | 3.745 | 4.780 | 4.875 | 4.210 |
| Dicalcium phosphate | 1.930 | 1.680 | 1.300 | 1.120 |  | 1.990 | 1.710 | 1.330 | 1.000 |
| Limestone | 0.970 | 0.880 | 1.200 | 0.710 |  | 0.990 | 0.880 | 0.790 | 0.660 |
| Salt | 0.535 | 0.525 | 0.500 | 0.475 |  | 0.505 | 0.495 | 0.475 | 0.450 |
| L-Lysine HCl | 0.090 | 0.096 | 0.154 | 0.183 |  | 0.092 | 0.100 | 0.170 | 0.197 |
| DL-Methionine | 0.341 | 0.322 | 0.349 | 0.286 |  | 0.335 | 0.313 | 0.317 | 0.260 |
| L-Threonine | 0.040 | 0.037 | 0.053 | 0.041 |  | 0.039 | 0.035 | 0.047 | 0.036 |
| L-Valine | - | - | 0.013 | 0.015 |  | - | - | 0.014 | 0.016 |
| Vitamin supplement^1^ | 0.145 | 0.127 | 0.100 | 0.082 |  | 0.145 | 0.127 | 0.100 | 0.082 |
| Mineral supplement^2^ | 0.145 | 0.127 | 0.100 | 0.082 |  | 0.145 | 0.127 | 0.100 | 0.082 |
| Choline chloride, 60% | 0.104 | 0.094 | 0.094 | 0.061 |  | 0.104 | 0.094 | 0.074 | 0.061 |
| Salinomicin, 12% | 0.036 | 0.036 | 0.036 | 0.036 |  | 0.036 | 0.036 | 0.036 | 0.036 |
| Antioxidant BHT^3^ | 0.050 | 0.050 | 0.050 | 0.050 |  | 0.050 | 0.050 | 0.050 | 0.050 |
| Avilamycin, 10% | 0.015 | 0.015 | 0.015 | 0.015 |  | 0.015 | 0.015 | 0.015 | 0.015 |

^1^Supplied per kg of vitamin supplement: Vitamin A 9,750 IU; Vitamin D₃ 2,470 IU; Vitamin E 36.6 IU; Vitamin B₁ 2.60 mg; Vitamin B₂ 6.50 mg; Vitamin B₆ 3.64 mg; Vitamin B₁₂ 0.015 mg; Pantothenic acid 13.0 mg; Biotin 0.091 mg; Vitamin K₃ 1.95 mg; Folic acid 0.91 mg; Nicotinic acid 39.0 mg. ^2^Supplied per kg of mineral supplement: iron 45.51 g; copper 9,480 mg; iodine 965 mg; manganese 66.47 g; selenium 287 mg; zinc 62.32 g. ^3^Butylated hydroxytoluene.**Supplementary Material 3.** Composition of diets from the 1983 Brazilian Tables program (BT 1983) and Cobb Nutritional program (Cobb 2022), formulated to meet the nutrient requirements of male and female broilers (as hatched), according to Rostagno et al. (1983) and Cobb-Vantress (2022), respectively.

| **Ingredients** | **BT 1983** | |  | **Cobb 2022** | | | |
| --- | --- | --- | --- | --- | --- | --- | --- |
|  | **1-27** | **27-42** |  | **1-12** | **12-27** | **27-39** | **39-42** |
|  |  |  |  |  |  |  |  |
| Corn, 7.88% | 59.748 | 63.354 |  | 53.533 | 59.490 | 63.251 | 67.952 |
| Soybean meal, 46% | 34.401 | 30.253 |  | 41.089 | 36.307 | 31.853 | 27.231 |
| Soybean oil | 1.780 | 2.685 |  | 1.020 | 0.630 | 1.565 | 1.615 |
| Dicalcium phosphate | 2.120 | 1.960 |  | 2.500 | 1.560 | 1.430 | 1.420 |
| Limestone | 0.920 | 0.880 |  | 0.520 | 0.740 | 0.700 | 0.700 |
| Salt | 0.370 | 0.360 |  | 0.370 | 0.370 | 0.370 | 0.370 |
| L-Lysine HCl | - | - |  | 0.113 | 0.131 | 0.143 | 0.157 |
| DL-Methionine | 0.166 | 0.142 |  | 0.313 | 0.289 | 0.265 | 0.228 |
| L-Threonine | - | - |  | 0.057 | 0.035 | 0.017 | - |
| L-Valine | - | - |  | - | - | - | - |
| Vitamin supplement^1^ | 0.145 | 0.100 |  | 0.145 | 0.136 | 0.120 | 0.090 |
| Mineral supplement^2^ | 0.145 | 0.100 |  | 0.145 | 0.136 | 0.120 | 0.090 |
| Choline chloride, 60% | 0.104 | 0.074 |  | 0.095 | 0.076 | 0.066 | 0.047 |
| Salinomicin, 12% | 0.036 | 0.036 |  | 0.036 | 0.036 | 0.36 | 0.036 |
| Antioxidant BHT^3^ | 0.050 | 0.050 |  | 0.050 | 0.050 | 0.050 | 0.050 |
| Avilamycin, 10% | 0.015 | 0.015 |  | 0.015 | 0.015 | 0.015 | 0.015 |

^1^Supplied per kg of vitamin supplement: Vitamin A 9,750 IU; Vitamin D₃ 2,470 IU; Vitamin E 36.6 IU; Vitamin B₁ 2.60 mg; Vitamin B₂ 6.50 mg; Vitamin B₆ 3.64 mg; Vitamin B₁₂ 0.015 mg; Pantothenic acid 13.0 mg; Biotin 0.091 mg; Vitamin K₃ 1.95 mg; Folic acid 0.91 mg; Nicotinic acid 39.0 mg. ^2^Supplied per kg of mineral supplement: iron 45.51 g; copper 9,480 mg; iodine 965 mg; manganese 66.47 g; selenium 287 mg; zinc 62.32 g. ^3^Butylated hydroxytoluene.
